# Science advocacy in political rhetoric and actions

**DOI:** 10.1007/s10669-022-09875-x

**Published:** 2022-08-19

**Authors:** Mark Quigley, Jeremy D. Silver

**Affiliations:** University of Melbourne, Parkville, VIC 3010 Australia

**Keywords:** U.S. Presidents, Science advocacy, Quantitative analysis, Science communication, Populism, American politics

## Abstract

*‘Science’* is a proportionately small but recurring constituent in the rhetorical lexicon of political leaders. To evaluate the use of science-related content relative to other themes in political communications, we undertake a statistical analysis of keywords in U.S. Presidential State of the Union (SOTU) addresses and Presidential Budget Messages (PBM) from Truman (1947) to Trump (2020). Hierarchical clustering and correlation analyses reveal proximate affinities between *‘science’* and *‘research’, ‘space’, ‘technology’, ‘education’*, and *‘climate’*. The keywords that are least correlated with *‘science’* relate to fiscal *(‘inflation’, ‘tax’*) and conflict-related themes (*‘security’, ‘war’, ‘terror’*). The most ubiquitous and frequently used keywords are *‘economy’* and *‘tax’*. Science-related keywords are used in a positive (promotional) rhetorical context and thus their proportionality in SOTU and PBM corpora is used to define fields of science advocacy (*public perception advocacy, funding advocacy, advocacy*) for each president. Monte Carlo simulations and randomized sampling of three elements: *language* (relative frequency of usage of science-related keywords), *funding* (proposed funding and allocated discretionary funding of science agencies), and *actions* (e.g. expediency of science advisor appointments, (dis-) establishment of science agencies) are used to generate a *science advocacy score (SAS)* for each president. The *SAS* is compared with independent survey-based measures of political popularity. A myriad of political, contextual, and other factors may contribute to lexical choices, policy, and funding actions. Within this complex environment ‘*science*’ may have political currency under certain circumstances, particularly where public and political perceptions of the value of science to contribute to matters of priority align.

**Supplementary Information:**

The online version contains supplementary material available at 10.1007/s10669-022-09875-x.

## Introduction

This research begins with the following question: *how frequently and variably do ‘science’ and related keywords appear in the rhetorical lexicon of standardized corpora delivered by leaders?* The leaders chosen to evaluate here are the Presidents of the USA, who regularly make decisions and communicate to the public on complex socioeconomic, political, and other issues. We consider rhetoric in its classical sense, articulated by Aristotle as “*means of persuasion in reference to any subject whatsoever*” (Rapp 2010). The corpora examined are the President’s annual State of the Union (SOTU) address and President’s Budget Message (PBM). A variety of statistical techniques are used to investigate the frequency and variability of keyword utility and to characterize correlations amongst keywords. The ‘related keywords’ are not defined a priori, but rather established through statistical analysis of keyword clustering. Intra- and inter-presidency comparisons are made using these results.

The research then queries, *can a metric for science advocacy be produced, and how does this relate to independent measures of political success?* Statistical approaches are used to combine metrics of science-related language, funding, and actions into a science advocacy score (*SAS*). The *SAS* is compared to mean approval poll ratings and a political greatness metric. The study is used to place science-related rhetoric and actions within a broader societal–political context, full of adjacent, interacting and/or competing themes that may emerge, escalate, and descend in objective, and/or perceived importance, at varying spatial and temporal scales. We explore the thesis that ‘*science advocacy*’ may be used as rhetorical tool for reflecting values and beliefs and may have political advantage in some circumstances.

In undertaking this research, we first acknowledge that ‘*science*’ as a discrete entity may not be unitary, comprehensive, collective, and even readily identifiable within the complex environment of political decision-making and actions (Guston 2010). We adopt the definition of “science” from the Science Council (2022) (https://sciencecouncil.org/about-science/our-definition-of-science/): “*the pursuit and application of knowledge and understanding of the natural and social world following a systematic methodology based on evidence*”. Whilst science inputs can inform many diverse decisions and policies, these ubiquitously reside alongside other relevant beliefs- and values-driven inputs, some of which may be prioritized above science inputs, and some of which might inform the way in which science is utilized in decision-making (Gluckman 2014; Quigley et al. 2019a, b). As stated by John Gibbons, science advisor to Pres. Clinton (from Pielke Jr. and Klein 2009):

“…science is not an overarching national goal for the President. It is only as it serves to help achieve these larger goals that science takes its place in the crown of important activities for the president”

Science may be pluralistic and partial (Guston 2010), particularly on matters where divergent scientific opinion is prominent. Some aspects of science exhibit strong partisan and ideological polarization (e.g. *climate change*, where 94% liberal Democrats believe that climate change is a major threat, compared with 19% conservative Republicans) (Kennedy and Hefferon 2019). Political polarization over science may be associated with *psychological science rejection* (implicit disregard for scientific facts that are inconsistent with one’s political identity) and/or *ideological science rejection* (adherence to a political ideology that explicitly contests science) (Rekker 2021). From their position of influence, U.S. Presidents may influence public perceptions (positively or negatively) of the value and utility of science and other priories, through lexical choices in communications (e.g. Cohen 1995; Gelderman 1995) and the relative status of different priorities in federal funding budgetary requests (e.g. Mervis 2017). President and aspiring presidents may frame science as a fallible entity,

“I don’t think science knows, actually” [with reference to climate change]

Fmr. President D. Trump, California Wildfire Briefing 14 September 2020 or as a symbol of truthfulness and trust,

“And I believe in Science” Hillary Clinton, July 28 2016 [Democratic Party nominee for U.S. Presidency, with reference to comments by then-Republican Party nominee Donald Trump on climate change]

“I’ve always said that the Biden–Harris administration, we’re going to lead, and we’re going to lead with science and truth; we believe in both” President-elect J. Biden 16 January 2021 [Democratic Party, pro-science rhetoric at announcement of the new administration’s scientific advisers]

and way of ‘thinking’ and ‘knowing’,

“Now and in the years ahead, we need, more than anything else, the honest and uncompromising common sense of science. Science means a method of thought. That method is characterized by open-mindedness, honesty, perseverance, and, above all, by an unflinching passion for knowledge and truth. When more of the peoples of the world have learned the ways of thought of the scientist, we shall have better reason to expect lasting peace and a fuller life for all.” Pres. Harry S. Truman, Address to the Centennial Anniversary AAAS Annual Meeting (1948)

**Fig. 1:**
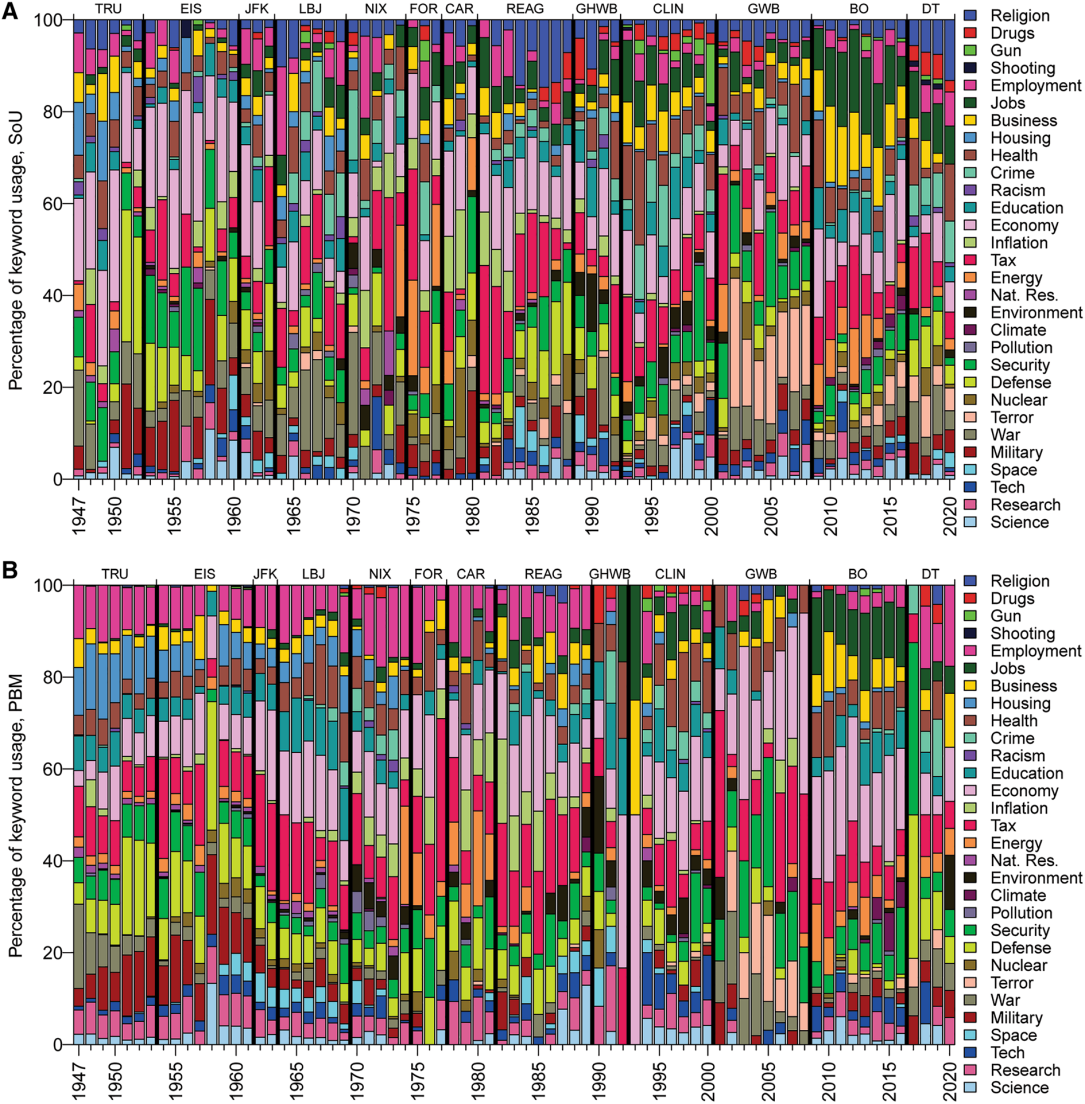
Keyword utility in presidential messages. **A** Time series of individual keyword utility as a % of total keywords in individual presidential State of Union (SOTU) addresses. Vertical-thick black lines denote presidential transitions. **B** Time series of individual keyword utility as a % of total keywords in individual PBM; line and colour labels as in (**A**)

Despite major changes in rhetorical styles, communication technologies (that have changed and diversified the media landscape), predominant methods of communication, and characteristics of the audience (Bennett and Iyengar 2008; Scacco et al. 2018), ‘science’ has remained a persistent entity in presidential communications through time (Fig. 1). Whether science-centric rhetoric has true political currency remains an open question.

In this study, we do not seek to disentangle the complexities of how scientific information is sought by, and considered, in presidential communications and policy making. Readers interested in this are encouraged to consult Pielke and Klein (2010) and the numerous references cited therein. Instead, we seek to develop objective measures for how U.S. Presidents advocate for science based on their lexical choices and actions. This is a challenging task.

Interviews with presidentially appointed chief science advisors reveal an environment where science and politics are endemically intermingled, where presidential behaviours appear to be variably technocratic, indifferent, and/or contradictory, and where communications between scientists and governing agents have become increasingly specialized and hierarchical (Pielke and Klein 2009; Launius and McCurdy 1997). The context of presidential communications and actions relating to science varies greatly in time and space and is important; emergent issues may enhance or diminish opportunities for science advocacy. Randomized selection and amalgamation of discrete pieces of pro- or anti-science evidence may be subject to various forms of sampling, confirmation, and selection biases.

U.S. Presidential communications typically cover a vast range of subjects, including commentary pertaining to actions, policies, and opinions on diverse and constantly changing social, economic, political, technological, and defence-related issues within a complex political ecosystem (Edwards and Howell 2009). Extensive research focuses on many aspects of presidential communications, including the transitory environment and context within which communications are made (e.g. Scacco et al. 2018), interactions between political rhetoric and the media (e.g. Herbst 2012), policy (e.g. Beasley 2010), power and influence (Campbell and Jamieson 2008), challenges (Denton 2000), and other rhetoric-related aspects (e.g. Gronbeck 1996; Stuckey and Antczak 1998; Kernell 1986; Tulis 1987; Medhurst 2008; Hart 1987).

SOTUs and PBMs are proxy measures of presidential priorities that have a relatively consistent format in approximate speaking duration (SOTU average = 53 min and 50 s, standard deviation ± 14 min; from Pres. Johnson to Trump), communication method (SOTU predominantly orated, PBM written), and audience (SOTU in person to the joint session of the United States Congress, transmitted to the public via the media). We suggest that these attributes make these the most standard and robust corpora for reducing bias in inter-president comparisons; it could be argued that sampling of any of the many other presidential communications (such as commemoration speeches and announcements of new initiatives) could introduce sampling bias, and other interpretive biases associated with the more specialized target audiences of the communications, variations in context, and other factors.

The SOTU gives “*to the Congress Information of the State of the Union, and recommend(s) to their Consideration such measures as he shall judge necessary and expedient*” (U.S. Constitution, Article II, section 3, Clause 1 1787). This provides an opportunity for the President to publicly advocate on priority issues, including those that may be informed by science, to Congress, the media, and a large public audience. The SOTU is generally accepted as the best means for assessing the president’s policy agenda (e.g. Cohen 1995, 1997; Kessel 1974; Light 1998; Oliver et al. 2011) and thus the endorsement of science in major public communications may considered a form of public science advocacy.

The PBM is the leading executive statement that accompanies the annual presidential budget request to Congress and provides insights into presidential budgetary constraints and philosophies, including advocacy for funding priorities (e.g. https://www.whitehouse.gov/wp-content/uploads/2019/03/ap_1_introduction-fy2020.pdf; https://www.everycrsreport.com/reports/R43163.html) such as federal research and development (R&D) funding for executive departments and independent agencies (Office of Management and Budget 2019). In addition to budget request, Presidents can make discretionary funding and organizational decisions (Sargent Jr. and Shea  2014; Lewis 2017) that may, in part, provide coarse proxies for how they value science relative to other priorities. The budget process is identified as one of the most important avenues through which scientists engage with the President (Pielke Jr. and Klein 2009),

“Most of the decisions that really have technical content get made within the government agencies at a level far below the White House. And it’s only rarely that science issues, or issues with technical content, actually come up to the White House for decisions or for policy direction change, but probably the most common way they come up is in the budget process and that's where a lot of the discussions that I have with my colleagues takes place.” John Marburger (science advisor to George W. Bush; as quoted in Pielke Jr. and Klein 2009)

We consider that presidential proposals to establish new science agencies, appropriate discretionary funds to science, and increase federal funding to science agencies may be broadly considered as a form of science funding advocacy. As final federal budgetary appropriations are ultimately decided by the U.S. Congress and may not reflect the budgetary recommendations of the President, we thus focus on presidential intent (rather than final science funding outcomes) in this analysis. We acknowledge that linguistic, financial, and structural reorganization decisions are likely to be strongly influenced by political factors, including partisanship; the potential underlying motives for science advocacy are briefly discussed but not investigated in detail here.

The data-driven approach undertaken here presents an objective, reproducible metric that is by no means perfect or exhaustive. Our metric intends to complement other types of analyses aimed to investigate science advocacy within the complex socio-political sphere of the U.S. Presidency (Pielke Jr. and Klein 2009) and stimulate continued research into role of science and affiliated themes in political rhetoric and actions.

## Materials and methods

### Keyword counts

**Fig. 2:**
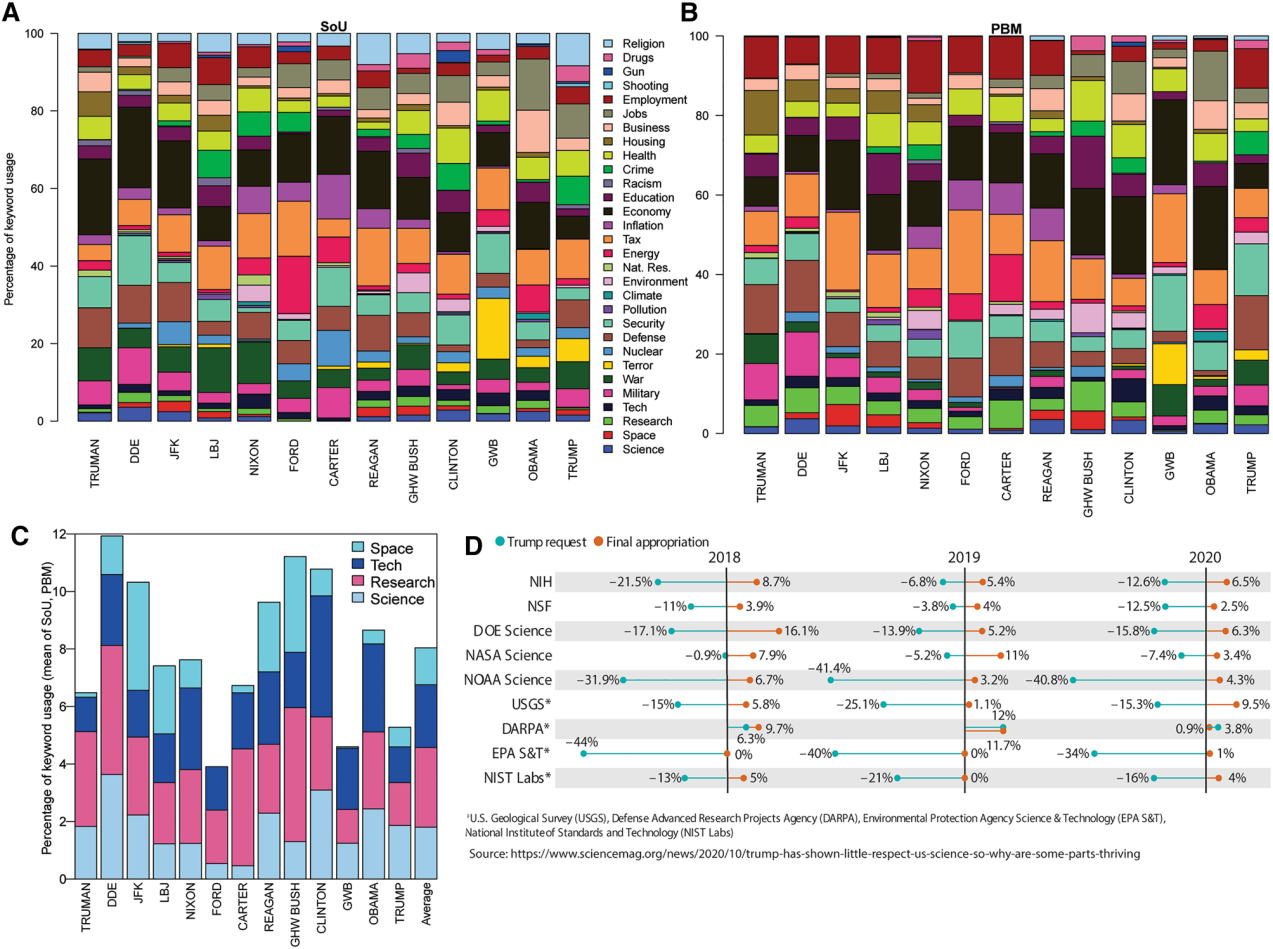
**A** Average keyword average utility and science advocacy in SOTU, by president. **B** Average keyword average utility and science advocacy in PBM, by president. **C** Combined (SOTU and PBM) science-related keyword utility (see Fig. 3 for clustering analysis) by president. **D** Example of U.S. Presidential Budget Message requests and Congress-approved final appropriations for major science agencies as a % of the previous year’s Congress-approved final appropriations. From 2018 to 2020, Trump proposed funding reductions for almost all science agencies but final federal budgetary appropriations resulted in increased funding. See https://www.science.org/content/article/trump-has-shown-little-respect-us-science-so-why-are-some-parts-thriving for further commentary

Statistical analyses of keywords and keyword groupings provide objective methods for comparing lexical salience between texts (Baker 2004; Bestgen 2018). Transcripts for SOTUs (*n* = 71) and PBMs (*n* = 80) from Truman (1947) to Trump (2020) were obtained from the American Presidency Project (http://www.presidency.ucsb.edu/) and FRASER digital library (https://fraser.stlouisfed.org/title/54). SOTUs and PBMs were read in detail to define transcendent topics of presidential communications. From these initial analyses, a suite of frequently used keywords (‘*science’, ‘technology’, ‘research’, ‘space’, ‘environment’, ‘economy’, ‘energy’, ‘natural resource’, ‘employment’, ‘jobs’, ‘housing’, ‘inflation’, ‘education’, ‘tax’, ‘health’, ‘business’, ‘crime’, ‘terror’, ‘gun’, ‘drugs’, ‘religion’, ‘shooting’, ‘military’, ‘research’, ‘security’, ‘climate’, ‘space’, ‘defence’, ‘nuclear’, ‘war’, ‘racism’, ‘pollution’*), including their bound morphemes, derivatives, and related words were identified (Supplementary Information Tables S1, S2). Keywords were counted in all presidential communications using automated scripts (Silver 2019) and manually cross-checked against SOTU and PBM transcripts for accuracy and context. Keyword frequencies were measured as a percentage of total keyword frequencies to normalize for large variations in total word counts (SOTU total word counts = 1080 ≤ *n* ≤ 9183; PBM = 294 ≤ *n* ≤ 30,140). Only orally delivered SOTUs were analysed to reduce potential bias arising from cross-comparison of different communication methods (Linnell 2004). Word counts for presidential communications are provided as a Supplement to this article. Individual keyword counts as a % of total keyword counts in SOTUs and PBMs are presented in Fig. 1A and B, respectively. Figure 2 presents average science keyword usages in combined SOTU and PBMs for each president.

### Dimension reduction and clustering analysis

**Fig. 3:**
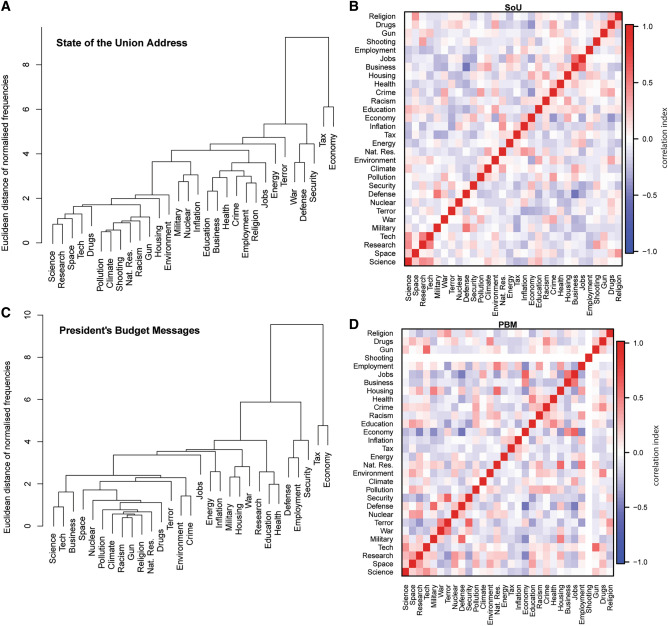
Clustering and correlation analyses of keywords in presidential messages. **A** Dendrogram of keywords in SOTUs. **B** Correlations between pairs of keywords, comparing their mean % usage in SOTUs. **C** Dendrogram of keywords in PBMs. **D** Correlations between pairs of keywords, comparing their mean % usage in PBMs. ‘Shooting’ did not appear in PBMs and is shown as having zero correlations

Unsupervised clustering was used to investigate keyword frequency data (Kaufman and Rousseeuw 1990). The averaged % keyword usage for each president was normalized across Presidents to have mean zero and unit variance; this was done separately for SOTUs and PBMs. Agglomerative hierarchical clustering was then performed on the Euclidean distance between columns (each column represented one keyword), agglomerating clusters by assuming distances to between a cluster and node (keyword) to be the furthest distance between that the outlying node and the nodes within the cluster. This yielded a clustering over keywords (Fig. 3A, C).

The y-axis of the keyword dendrograms (Fig. 3A, C) shows the “dissimilarity” between pairs of keywords. The dissimilarity between a pair of keywords was taken to be the Euclidean distance between the vectors of normalized frequencies, comprising one element per president (averaging over the keyword percentage usages across all their messages of a given type). The dendrogram was incrementally constructed by agglomerating pairs of nodes and/or clusters; once agglomerated, distances between the new cluster and other nodes or clusters were taken to be the maximum distance in the comparison in question (this is termed agglomeration via ‘complete linkage’).

Heatmap plots (Fig. 3B, D) characterize the linear correlations between the normalised counts for the individual keywords (highest possible correlation = 1, lowest possible correlation = − 1). Keyword clustering and correlation plots delineate ‘science-related’ keywords that are most closely associated (i.e. proximate) with ‘*science*’; these are ‘*research’, ‘technology’, ‘space’*, and to a lesser extent ‘*climate*’ and ‘*education*’. Keywords that are least correlated with *‘science’* relate to fiscal *(‘inflation’, ‘tax’*) and conflict-related challenges (*‘security’, ‘war’, ‘terror’*). A sample of contextual time series data is presented in Supplementary Information Fig. S1.

### Science advocacy plots

**Fig. 4:**
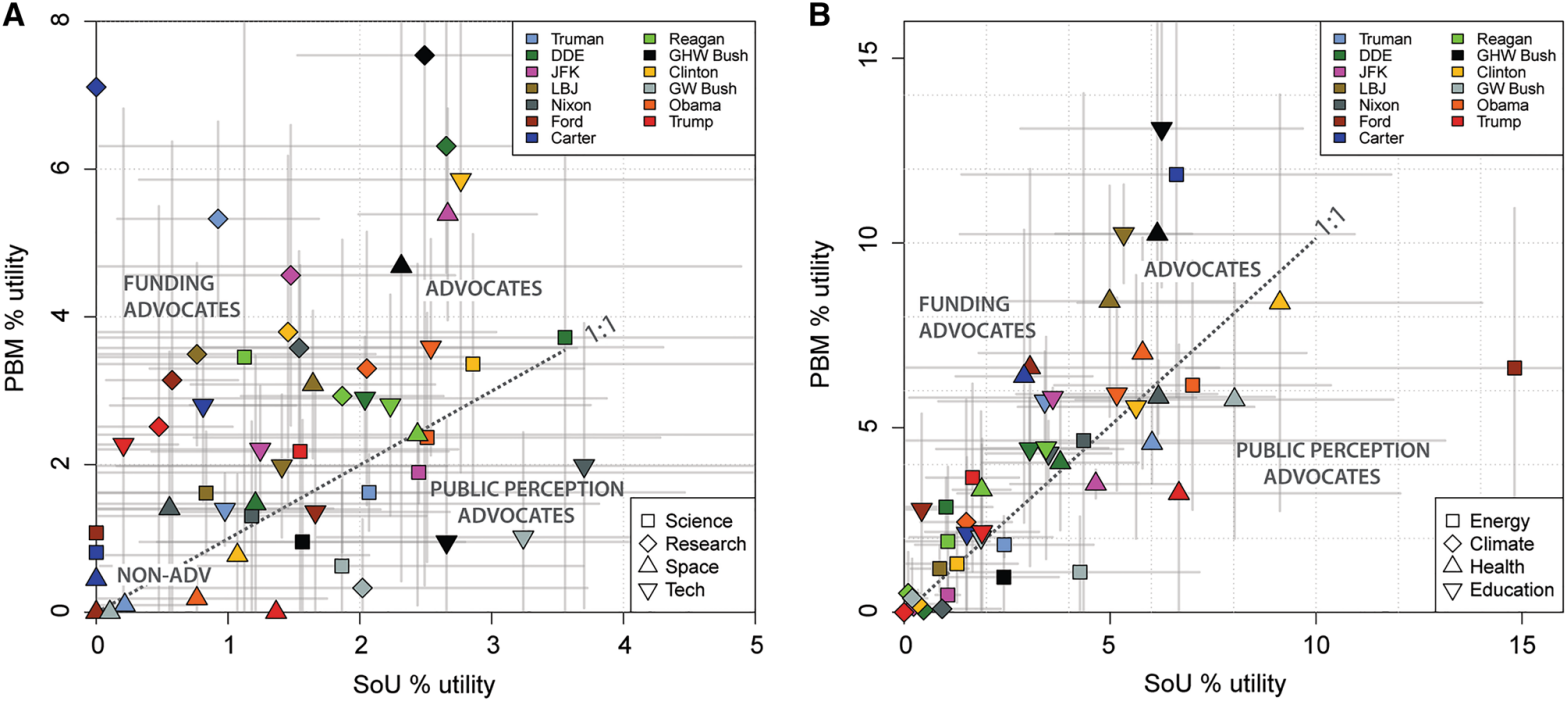
Keyword % usage in the two message types and science advocacy. **A** Comparison of average % usage of individual, most proximate (i.e. shortest Euclidean distances, largest correlation indices in Fig. 3) science-related keywords in SOTUs, and PBMs for each president. Symbol shape corresponds to keyword, and symbol colour corresponds to president. Labelled ‘advocacy fields’ correspond to higher-than-average science keyword utility % in SOTUs only (‘public perception advocacy’), higher-than-average science keyword utility % in PBMs only (‘funding advocacy’), higher-than-average science keyword utility % in PBMs and SOTU addresses (‘advocacy’), and lower-than-average science keyword utility % in PBMs and SOTU addresses (‘non-advocacy’). Advocacy fields are intended for conceptual purposes; boundaries between each field are not distinctly defined. **B** Comparison of average % utility of other science-related keywords in SOTU addresses and PBMs for each president. The ‘climate’ data points (Fig. 2B) amalgamate ‘climate’, ‘environment’, ‘natural resources’, and ‘pollution’ data, since these terms are commonly topically grouped in PBMs and SOTUs. As with (**A**), the selected keywords have the shortest Euclidean distances and largest correlation indices in Fig. 3

Selected keyword average % usages for individual presidents in SOTUs vs. PBMs are presented in Fig. 4. The fields are defined as (i) ‘*Public Perception Advocates*’: higher-than-average % SOTU counts, low % PBM counts, (ii) ‘*Funding Advocates*’: higher-than-average % PBM counts, low % SOTU counts, (iii) ‘*Advocates*’: higher-than-average % SOTU counts and % PBM counts), and (iv) ‘*Non-advocates*’ (low % SOTU counts and low % PBM counts). The boundaries between advocacy fields are indicative, rather than representing substantive thresholds.

### Presidential SAS and political popularity

**Fig. 5:**
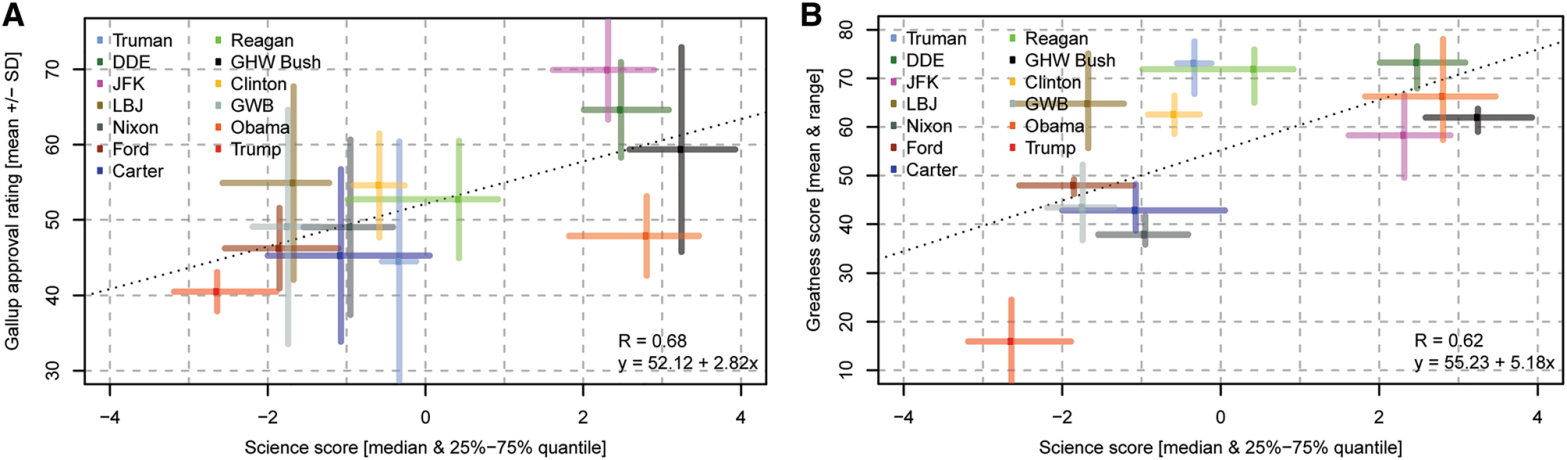
Presidential approval, presidential greatness, and *SAS*. **A** Presidential mean daily Gallup poll approval rating vs. *SAS* (“science score”). Mean daily approval rating determined by interpolating data between successive polls assuming linear inter-poll trajectories. Vertical error bars represent 1 standard deviation for all discrete polling data results for the president listed. Coloured horizontal error bars represent the interquartile range of the sub-sampled science scores. The equation for the linear best fit line is shown, as is the Pearson correlation (R). **B** 2018 Qualtrics Presidents & Executive Politics Presidential Greatness Survey mean rating vs. science score. Vertical error bars represent mean ratings differentiated by party of survey respondents. Other elements of the plot (line of best fit, correlation, horizontal error bars) are as for Fig. 5A

The time series of Gallup poll approval rating % data from 29 May 1945 (Truman) to 16 June 2019 (Trump) are shown in Supplementary Information Fig. S2. Gallup reports a ~ 3% uncertainty in up-scaling results from the sampled population (~ 1000 people) to the larger population and the survey is intended to represent. There is uncertainty in using the mean approval rating value for a president for comparative purposes, partly because of the large variations in the relative timing and frequency of the polls (conducted up 100 days apart with significant variability over 1938–2008, then daily over 2009–2017, then weekly in 2018). To address this aspect, we fitted a smoothing spline through the approval rating data for each president, used this to interpolate to daily frequency, and then calculated a mean Gallup poll approval rating (*y*-axis; Fig. 5A) from the daily averages.

The 2018 Presidential Greatness poll results shown on the y-axis in Fig. 5B (Vaughn and Rottinghaus 2018) are presented as a mean and range. This metric was established via a presidential greatness survey of current and recent members of the Presidents and Executive Politics Section of the American Political Science Association conducted in 2017–2018. Respondents were asked to rate each president on overall greatness on a scale from 0 to 100 (0 = failure, 50 = average, and 100 = great); 170 usable responses were tabulated. The Presidential Greatness poll data are presented as the mean value ratings from survey respondents identifying as the Democrat, Republican, and Independent/Other. The survey respondents were skewed towards Democrat and Independent-affiliated voters beyond the US average, thus we summarized the Presidential Greatness poll as a weighted mean of the three ratings, with weightings taken as the average over 2004–2019 of Gallup Polls of party affiliation across US voters (https://news.gallup.com/poll/15370/party-affiliation.aspx); these indicate 29.5% Republican, 32.9% Democrat, and 37.6% Independent. The range presented for the Presidential Greatness score was taken as the minimum and maximum greatness score amongst the three party/non-aligned groups.

For Fig. 5A and B, the ‘*SAS*’ is based on three components: actions, funding, and language. Each of these categories comprised a series of factors that are outlined below. In developing this method, we recognize that there is no objective measure of a President’s support for science (or lack thereof) that is free from subjective and interpretive bias. Others approaching this topic may assign different weights to these components and factors. Given this epistemological uncertainty, we considered a range of different possible realizations of the *SAS*, randomly sub-sampling the factors comprising the three components (*actions, funding, language*). One advantage of this approach is that it provides some insight into the uncertainty of the *SAS* (i.e. the range of possible views that may be adopted for a given President’s public support for science). In the manner defined below, there were 44,100 equiprobable science scores under this sampling scheme. Figure 5A and B shows the median and inter-quartile range of 5000 random samples (with replacement).

The ‘*actions*’ component was based on eight factors (Supplementary Information Table S3): (1) the presence of a science representative within the Executive Office of the President, (2) inter-agency coordination organizations, (3) science-related advisory committees, (4) independent science-related agencies, (5) multi-agency science-related initiatives, (6) other non-defence Federal research agencies, (7) defence research agencies, and (8) the promptness of the appointment of a director for the Office of Science and Technology Policy (OSTP). For each component, points were evaluated for establishment (+ 1), abolition (− 1), or continuation (0) of an agency or representative or for the last factor the appointment (+ 1) or otherwise (− 1) of a director of the OSTP within 100 days of assuming office. The total number of points was adjusted for time in office by dividing by the length of the presidency (in years). Each random sample selected four of these eight factors, and the resultant scores were normalized to have zero mean and unit variance. We acknowledge that more extensive lists of ‘anti-science’ actions are available (e.g. https://www.ucsusa.org/resources/attacks-on-science?_ga=2.145380413.863759956.1612999703-1619096890.1612999703) but that variations in the extent to which past presidents have been analysed following this approach preclude unbiased inclusion of these analyses into our research framework.

The ‘*funding*’ component was based on three factors: (1) changes to research and development funding as a percentage of discretionary outlays during tenure, (2) changes to non-defence research and development funding as a percentage of non-defence outlays, and (3) changes to funding for the OSTP. As with the ‘actions’ component, points were assigned for each factor for increases (+ 1), reductions (− 1), no change (0), and with half-points assigned for minor changes. Each random sample selected two of these three factors, with the resultant scores normalized as above. Because of the complexity of the U.S. Federal Budget (https://www.aaas.org/news/federal-budget-process-101), including incumbent’s revisions of budgets developed by their predecessors and complex inter-agency interactions, interpreting monetary assignments as a proxy for presidential priorities is challenging. OSTP funding is by no means a perfect proxy for the value a given president places on science advice but it is perhaps one of the simplest objective measures available as its administering organization is the Executive Office of the President. In theory, a president that values science advice from the OSTP might assign a higher % of discretionary funding to this office than a president who does not. The history and contributions of OSTP and its affiliates (President’s Council of Advisors on Science and Technology; National Science and Technology Council) and their predecessors are available at https://obamawhitehouse.archives.gov/administration/eop/ostp/about, Pielke Jr. and Klein (2009), and Sargent Jr. and Shea (2014).

The ‘*language*’ component was based on the SOTU and PBM word counts. Eight keywords were considered (‘science’, ‘research’, ‘space’, ‘tech’, ‘energy’, ‘climate’, ‘health’, ‘education’) on the basis of contextual reading of the corpora and the results of the hierarchical clustering analyses. Each random sample included a selection of four of these keywords. Also selected at random was the source: the SOTU only, the PBM only, or both the SOTU and PBM. The counts of each keywords were converted to the average percentage keyword use for each president across messages. We then took the ratio of each President’s average percentage keyword use to the average percentage keyword usage across presidents. This ratio was normalized in the same manner as the two components described above.

## Results

### Keyword usage in presidential communications

The most frequently used keywords in SOTUs and PBMs (Fig. 1) are ‘*economy*’, ‘*tax*’, ‘*security*’, and ‘*defence*’. The least frequently used words are ‘*shooting*’, ‘*climate*’, ‘*pollution*’, ‘*racism*’, and ‘*gun*’. Some keywords show significant temporal trends in usage that transcend presidential changes, such as ‘*jobs*’ and ‘*health*’ (average ascendency through time), ‘*housing*’, ‘*employment*’, and ‘*natural resources*’ (average diminishing use through time), and ‘*inflation*’ (ascendency to highest usage in the 1970s to early 1980s followed by descent).

The % usage of the most proximate science-related keywords (‘*science’, ‘research’, ‘technology’, ‘space’*; as defined from the hierarchical clustering) has no statistically significant temporal trends and is highly variable about mean % usage (coefficient of variation > 1). ‘*Science*’ and *‘technology*’ are the most frequently used keywords in SOTUs and ‘*research*’ is the most used keyword in PBMs. ‘*Science*’ usage varies significantly through time (0 to > 10% usage) but is ubiquitously used in a positive, promotional sense (i.e. there is no evidence in SOTUs or PBMs of anti-science rhetoric). Positive spikes in usage are observed for many presidents. Some can be confidently interpreted as contextual evidence concurrent with the emergence of important events in U.S. science policy (e.g. the establishment of the National Science Foundation in 1950 (Truman; SOTU), the ascendency of science advice to priority status in the White House following the 1957 Soviet Union launch of Sputnik (Eisenhower; SOTU and PBM in 1958). Some presidents primarily speak of ‘*science*’ as a valued entity in health, educational, and technological contexts (Clinton, Obama), others to advocate for funding (Reagan), and others to recount U.S. historical achievements (Trump 2019 SOTU). Eisenhower, Clinton, and Obama are the highest average users of ‘*science*’; Ford and Carter did not mention ‘*science*’ in any SOTUs and are amongst the lowest users in PBMs. ‘*Research*’ and ‘*technology*’ commonly positively correlate with ‘*science*’ usage, although exceptions exist (e.g. Carter; PBM). ‘*Space*’ usage is highest during the height of the “Space Race” (ca. 1960 to 1970), re-emerges in usage (concomitant with increases in ‘*defence*’ usage) during Reagan’s Strategic Defence Initiative or “Star Wars” agenda (Krug 2004), and was prominent during GHW Bush’s Space Exploration Initiative in 1989, before declining to minimal utility. Presidents considered to have advocated most strongly for NASA funding in Congress (JFK, Reagan, GHW Bush) (Conley and Cobb 2012) are clear outliers in ‘*space*’ usage in PBMs. The largest combined users for science-related keywords are Eisenhower, GHW Bush, Kennedy, and Clinton; the lowest are Ford, GW Bush, and Trump (Fig. 2). There is complementary evidence to suggest that the most frequent users of science-related keywords in SOTUs and PBMs also placed value on science in other communications,

“Love of liberty means the guarding of every resource that makes freedom possible—from the sanctity of our families and the wealth of our soil to the genius of our scientists.” Dwight D. Eisenhower, 20 January 1953 [Republican Party, First Inaugural Address]

Figure 4 examines the relationships between keyword use in SOTU vs. PBM for science-related keywords (see also Fig. 3). Science keyword % usage for SOTU vs. PBM is plotted by president and used to estimate generalized fields of science advocacy (‘public perception advocates’, ‘funding advocates’, ‘advocates’, and ‘non-advocates’ = non-adv). In Fig. 4A, approximately 2/3 of the data points reside above the 1:1 line, indicating the most proximate science-related keywords are typically used more frequently in PBMs than SOTUs (i.e. Presidents tend to act more as funding advocates than public perception advocates on these topics). The most frequent users of science keywords (i.e. science ‘advocates’) are Eisenhower, Kennedy, Clinton, GHW Bush, and Reagan. GW Bush used science keywords in SOTUs more frequently than many of his compatriots, but rarely used them in PBMs (‘public perception advocate’). Several presidents (e.g. Carter, Truman, Ford, Johnson) advocated significantly more for ‘research’ in PBMs than SOTUs (i.e. ‘funding advocates’).

Figures 1, 2, and 4 present insights into presidential advocacy for a diversity of priorities, some of which could be considered science related, depending on context. Notable advocates are Carter and Ford for ‘*energy*’, Clinton for ‘*health*’, Johnson for ‘*education*’, and Nixon and Clinton for ‘*climate*’ (including ‘*environment*’, ‘*natural resources*’, and ‘*pollution*’; see Fig. 2 caption). Obama, Clinton, and Nixon are the largest advocates of the potentially science-related keywords shown in Fig. 4B; Eisenhower, Reagan, Truman, and Trump are the least. Relationships between keyword usage and historical presidential actions and agendas are addressed in the Discussion.

Keyword % usage of ‘*war*’ is highest in Truman’s communications following World War II, during the peak of the Vietnam War (Johnson and Nixon), and in the lead up to and commencement of military action during the Iraq war (GHW Bush) and the ‘war on terror’ (GW Bush) (See Supplementary Information Fig. S1). Excluding its heightened usage during the Korean War and escalation of Cold war tensions during Truman’s tenure, the keyword ‘*defence*’ has been most frequently used outside of periods of major military conflicts and may reflect the emergence of real or perceived international threats and presidential priority initiatives and values (e.g. Eisenhower’s communications may reflect his military-based employment history and value system, the Cuban Missile Crisis during JFK presidency, nuclear threats during Carter presidency, advocacy for the Strategic Defence Initiative during Reagan presidency, advocacy for defending America’s borders during Trump presidency). ‘*Military*’ usage was highest in the Truman and Eisenhower presidencies and spiked during denouncements of emerging Soviet military action in Afghanistan by Carter, during intermittent military engagements during the Reagan presidency and at the commencement of military conflicts in Afghanistan and Iraq during the GW Bush presidency.

‘*Economy*’ is the most utilized keyword for both SOTU and PBMs. The two-year moving average % usage typically fluctuates between 10 and 20% for all presidents except for Trump. Temporal variability in the use of ‘*economy*’ is complex. There is a tendency for ‘*economy*’ to be used more frequently during times of stronger domestic economic performance (higher % annual change in real GDP per capita) and less during economic recessions, suggesting that a large fraction of its usage is primarily related to gaining political advantage from economic prosperity or recovery rather than to advocate for economic change during reduced economic performance. Divergent usage of this keyword is also evident in the different message formats; during the 2007–2009 global financial crisis GW Bush used ‘*economy*’ frequently in PBMs to advocate for economic stimulus, but reduced usage in SOTU presumably due to the potential for adverse political ramifications of further escalating this issue in the public eye, whilst Obama particularly increased % usage of ‘*economy*’ in early SOTU addresses to advocate for economic stimulus and policy reform and to gain political advantage from economic recovery. The % usage of ‘*inflation*’ tends to correlate with % annual changes in consumer price index (CPI); peak usage in SOTUs and PBMs is concurrent with large CPI increases (and more frequent recessions) during the 1970s to early 1980s.

‘*Science*’ and ‘*research*’ commonly exhibit positive correlations (as evident from Eisenhower, GHW Bush, Clinton and Obama communications), which are sometimes accompanied by increases in the usage of ‘*technology*’ (Nixon, Clinton, Obama) and ‘*space*’ (Reagan, Eisenhower). This is consistent with results from the clustering analyses. There is a tendency towards more less frequent usage of ‘*science*’ and ‘*research*’ during economic recessions and periods of heightened inflation and more frequent usage during periods of economic stability or growth (Fig. S1). This is also evident in the strong negative correlations between science-related keywords and ‘*economy*’, ‘*inflation*’, and ‘*tax*’. There is a tendency for increased ‘*science*’ and ‘*research*’ usage outside of periods of military conflict. We hypothesize that ‘*science*’ and ‘*research*’, and to a lesser extent ‘*technology*’ and ‘*space*’, may be considered as optional linguistic components of political messaging, where their usage at a given time is highly subject to the prevalence of other non-optional socioeconomic and militaristic issues and presidential priorities. Put more brazenly, perhaps some leaders consider ‘science’ as a luxury item to feature more prominently in times of peace and prosperity, with reduced rhetorical usage when urgent economic and militaristic matters ascend in priority. Of the presidents with positively performing economies and declining or low levels of military engagement, Clinton was the most prolific user of science keywords and Trump the least.

### Science advocacy and political popularity

The behaviour of presidential polling data is reasonably well understood (e.g. Mueller 1973; Erikson et al. 2002; Eichenberg et al. 2006). Presidential polling data tends to show a ‘honeymoon’ period of elevated approval ratings following election or re-election, a subsequent decline approval rating with time (although this is not ubiquitous, e.g. Clinton), longer-term variations in polling trends (that could be related to economic performance, and/or involvement in costly wars with large accumulations of fatalities, for example, Truman decline during Korean War and Kennedy and Johnson declines during Vietnam war; Hibbs 2000) and episodic perturbations (i.e. ‘rally events’, such as surge in approval for GW Bush after declaration of the ‘war on terror’ following the 11 September 2001 terrorist attacks) (Eichenberg et al. 2006). Almost every president begins their tenure with sustained, elevated approval levels compared to their predecessor. Intra-presidency approval ratings commonly vary by > 30%. The presidents with the highest mean approval ratings are Kennedy, Eisenhower, GHW Bush, and Clinton; the lowest are Trump, Truman, and Carter. Truman, GW Bush, and GHW Bush have the highest standard deviation in mean approval rating. The presidential greatness survey scores (derived from Vaughn and Rottinghaus 2018 and modified for poll respondent party affiliation) are highest for Eisenhower followed by Truman and Reagan and lowest for Trump (lowest), Nixon, Carter, and GW Bush (Fig. 5B). The SAS are highest for GHW Bush, Obama, Eisenhower, and Kennedy and lowest for Trump, GW Bush, and Ford (Fig. 5). The SAS show a positive correlation with both the contemporary and historical approval ratings (Fig. 5).

## Discussion

Although political parties may sometimes take partisan approaches to science-related issues, presidential communications and actions relevant to science issues and funding may be challenging to analyse objectively and may be difficult to characterize as pro- or anti-science (Fisher 2013). To gain objective insights into presidential communications using a standardized framework, we analysed a uniform set of *corpora* (Presidential SOTU and PBMs) using uniform criteria (keyword counts) that are internally normalized to account for variations in *corpora* length (keyword as a % of total keywords used). Our objective was to undertake an objective analysis that is easily reproducible and immune from many potential forms of cognitive bias, partisanship, and heuristics (Kuklinski and Quirk 2000).

Several key observations pertaining specifically to the relative frequency of science keyword use in presidential communications warrant discussion here. There is no clear association between political party and the % usage of ‘*science*’, ‘*research*’, ‘*space*’, or ‘*technology*’ in either SOTUs or PBMs. Eisenhower (Republican), Kennedy (Democrat), GHW Bush (R), Clinton (D), and Reagan (R) are the most frequent users of science-related keywords, whilst Trump (R), Carter (D), Ford (R), and Johnson (D) are the least (Fig. 2A). All presidents have delivered at least one SOTU or PBM communication where each of the science-related keywords are less than the presidential average % usage. There is also no clear relationship between political party and other selected science-related keywords as shown in Fig. 4B. Care must be taken to view the collective of their messages to evaluate science advocacy during their presidency rather than focusing on a single message (hence keyword % usage averaged over messages was used in the PC analysis and clustering), and any suggestion that a specific presidential message enables characterization of a long-term prevailing political party view is not evidenced in these data (Fig. 1). An emergent pattern is that many presidents occupy an outlier-type position in at least one specific science-related keyword, and their linguistic advocacy for this theme is independently supported by their presidential actions. A prime example of this is Nixon, whose advocacy in the combined *‘climate’* + *‘environment’* + *‘natural resources’* + *‘pollution’* field (Fig. 4B) is consistent with his track record in environmental policy and advocacy that include establishment of the National Environmental Policy Act (1969), the Environmental Protection Agency (1970), the National Oceanic and Atmospheric Administration (NOAA 1970), the Clean Air Act (1970), Earth Week (1971), the Clean Water Act (1972), and the Endangered Species Act (1973). Clinton’s advocacy for *‘technology’* and *‘health’* is similarly supported by actions, including establishment of the Climate Change Technology Initiative (2000), the E-rate and the Technology Literacy Challenge Fund (1996), the National Nanotechnology Initiative (2000), and the Clinton Health Access Initiative (2002), amongst others (see Supplementary Information) (https://clintonwhitehouse5.archives.gov/WH/Accomplishments/eightyears-09.html). The absence of advocacy may also be supported by actions in some instances; for example, one of the lowest Presidential users of ‘*science’* and *‘research’* (Pres. Trump, particularly in 2017–2018) proposed large science budget cuts (Malakoff and Cornwall 2017; Mervis 2017) (Fig. 2D), delayed appointments of chief science advisors (Goldman et al. 2017), and dissolved science advisory councils (Sargent Jr. and Shea 2014). GW Bush was described as engaging in a “war on science” (Mooney 2005).

We cannot fully understand the extent to which science advocacy might be entirely politically motivated, or if some emergent issues demanded a role for science whether the President was interested in advocating for science or not. We acknowledge that once science institutions were established (e.g. the National Science Foundation by Truman in 1950) future presidents could not score advocacy points in this aspect, even if supportive of these science agencies, but could score advocacy points in PBM messaging and proposed funding for these agencies. The absence of advocacy (e.g. for science) may be a simple manifestation of attendance to more urgent priorities, even if a President had a personal and vested interest in science. Our probabilistic, resampling approach to the construction of a science score represents our best effort at trying to objectively address these types of potential interpretative concerns.

We endeavour to minimize potential bias associated with word selection, word omission, and weighting of words, actions, and funding (e.g. *should other keywords have been counted? Are the metrics used to calculate the SAS sufficient in volume and representation?*) by (i) applying the same criteria to all presidential communications wherever possible, so that an emphasis is placed on comparative analysis amongst the presidential cohort and (ii) applying Monte Carlo simulations and randomized sampling of possible combinations of language, funding, and actions metrics to develop SAS and associated error bounds for each president. Due to the low population of science-related keyword counts (25th and 75th quartile values of 0 ≤ *x* ≤ 4 counts in SOTUs with median values of 0–2), the data are highly sensitive to small fluctuations in usage. Multiple mentions of ‘*science*’ and science-related keywords in SOTUs, given the size of the attendant audience and competition to address many priorities, are assumed to represent an agenda that is linguistically distinct from one that does not mention these words.

The analysis of political language by automated content methods is generally intended to supplement, rather than replace, thoughtful reading, and contextual analysis of communications (Grimmer and Stewart 2013). A detailed analysis of the historical political and socioeconomic contexts of each keyword is well beyond the scope of this study (however, some contextual analysis is presented in the time series plots, see Supplementary Data). Instead, we focused primarily on science-related keywords and their most obvious relationships to other keywords. We acknowledge that many keywords that appear to have the highest divergence from science-related keywords (e.g. economy, security, defence, drugs) relate to issues that can be informed by science and could be lexically used within a science context, but our reading and interpretations of samples from the SOTU and PBM transcripts indicate that the clear majority are not. Instead, they are primarily used to communicate on socioeconomic, health, and/or foreign or domestic policy and security issues, for which the contextual relevance of science is commonly unstated. Whilst we acknowledge the same keyword may be used for different purposes, for example, to lobby support for political action on an emerging challenge (e.g. avoiding an ‘economic’ recession) or to claim success from measures taken for political benefit (e.g. a strong ‘economy’), science-related words are not used in an anti-science context. Some keywords may have multiple meanings (e.g. illegal ‘*drugs*’ as narcotics vs. ‘*drugs*’ as prescription medications; ‘*health*’ care vs. ‘*health*’ of the economy—see Supplementary Information); we identify this as an additional source of uncertainty in word data. Given the various assumptions and uncertainties inherent to this analysis, we caution against over-interpreting these results.

The foregoing invites consideration of Donald Trump’s science advocacy and comparison with that of his predecessors. Such an analysis is of benefit because Trump sought and realized political capital thorough populism that included rhetorical anecdotes (excluding SOTUs and PBMs) that could be considered negative towards science, scientists, and experts more generally. According to some analyses, Trump’s ‘impulsive’ ‘failure’ in response to COVID-19 pandemic (https://www.washingtonpost.com/elections/interactive/2020/trump-pandemic-coronavirus-election/), which included anti-mask sentiment, anti-science advice sentiment, and other populistic rhetoric aimed at diminishing the role of science and scientists, may have been a critical factor in his loss of the 2020 election (https://www.politico.com/f/?id=00000177-6046-de2d-a57f-7a6e8c950000). In important respects, Trump’s populism built on a scepticism towards scientific expertise and his elevation of instinct (his own) as the essential commodity in decision-making.

Trump ranked lowest in science keyword usage and science advocacy in the analysed SOTU and PBMs. Trump also proposed significant reductions in funding to almost every major governmental science agency throughout his presidency (Fig. 2D). Ironically, Congress countered these proposed funding reductions with funding increases to many U.S. science agencies (Fig. 2D). The National Institutes of Health (NIH), the biggest federal supporter of academic research, has increased its budget by 39% in the past 5 years despite budget cuts proposed by Trump, and the budget of the National Science Foundation (NSF) increased 17% from 2018–2020.

Numerous studies have investigated the ‘success’ of U.S. Presidents from diverse perspectives and using distinct proxy measures, including election success (Hibbs 2000), time in office and relationship to presidential greatness (Cohen 2003), success in Congress and legislation (Rogowski 2016; Barrett and Eshbaugh-Soha 2007), and success in supreme court appointments (Segal et al. 2000). Here we use simple polling-based metrics for success: average public approval rating and expert opinion-derived Greatness scores. Neither metric captures the complete and coherent picture of presidential success; Truman and Obama are amongst the least popular presidents in average approval rating but score amongst the highest in Greatness, for example. However, when the presidential cohort is considered *en masse*, there is in general a positive correlation between SAS and (i) approval rating and (ii) Greatness score. These relationships need not imply direct causation; other economic indicators (e.g. growth of real disposable personal income per capita) and cumulative military fatalities are more indicative predictors of popularity (Hibbs 2000; Eichenberg et al. 2006). Presidential Greatness could hardly be uniquely attributed to science advocacy given the relatively low use of science keywords in presidential communications, the small (typically < 1.2%) of federal funding for research and development as a % of gross domestic product (https://www.aaas.org/programs/r-d-budget-and-policy/historical-trends-federal-rd), and the near-continuous emergence of domestic and international issues that feature more prominently in U.S. political and public discourse. However, scientific and technological achievements rank 3^rd^ behind America’s armed forces and its history in a survey of nationalist pride (96% of respondents stated they were proud of these achievements) (Bonikowski and DiMaggio 2016). Is it possible that Trump’s 2019 SOTU, where ‘science’ usage was associated with a historical pride-in-achievement context and was anomalously high relative to his preceding SOTU’s and sought advantage from this relationship? Could future presidents and political strategists seek to capitalize on this? It remains possible that the prioritization of science-related issues within the complex environment of democratic politics, regardless of the motive or context, may yield subtle political advantages that are not yet well captured or understood. Indeed, President Biden’s early ‘pro-science’ agenda has included rapid action on COVID-19, climate change, and appointment of scientists into key roles in his administration (https://www.scientificamerican.com/article/biden-elevates-science-in-week-one-actions/). Regardless of the potential causal chains between science and political success, we hope this paper will assist in stimulating further research in this area.

Our analysis provides intriguing insights into the utility and variations in how science features in presidential communications. Different methodologies (e.g. topic analyses, text dispersion keyness—Grimmer and Stewart 2013; Jacobi et al. 2016; Egbert and Biber 2019) and other political communications (Grimmer 2010) could be used to further interrogate the results presented herein. We hope this study contributes quantitative evidence to inform contemporary debates on issues, such as presidential attitudes towards science (Fisher 2013; Lane and Riordan 2018), and contributes to other studies of U.S. Presidents (e.g. Thoemmes and Conway III 2007; Watts et al. 2013; Roediger and DeSoto 2014).

## Conclusion

- *‘Science’* and related keywords (*research, space, technology*) constitute a proportionately small (ca. 5–10%), but persistent element in the rhetorical lexicon of U.S. Presidents from Truman to Trump, transcending time and political party.
- Fiscal terms (‘*economy*’, ‘*tax*’) are the most used keywords in presidential communications; *inflation’, ‘tax’, ‘security’, ‘war’,* and *‘terror’* are the keywords least correlated with science keywords
- ‘*Science*’ and related keywords are used in a positive (promotional) rhetorical manner and thus their proportionality in SOTU and PBM corpora is a proxy measure for science advocacy
- Monte Carlo simulations of U.S. Presidential language, funding proposals and allocations, and actions are used to estimate a *SAS* for each president that is compared with independent measures of political success
- Positive correlations between the *SAS* and measures of presidential popularity and greatness do not constitute causation, but suggest that science advocacy could have political currency in some contexts, as potentially evident in the most recent U.S. Presidential election campaign (Pres. Biden)

## Supplementary Information

Below is the link to the electronic supplementary material.

Supplementary file1 (TXT 36 KB)

Supplementary file2 (TXT 40 KB)

Supplementary file3 (XLSX 22 KB)

Supplementary file4 (DOCX 1278 KB)
