# Supplementary material for: Science advocacy in political rhetoric and actions
Source: Environ Syst Decis. 2022 Aug 19;42(3):462–76. doi: 10.1007/s10669-022-09875-x (PMC9389511; doi:10.1007/s10669-022-09875-x)
Supplement: Supplementary file 4 — Supplementary file4 (DOCX 1278 KB) [file 10669_2022_9875_MOESM4_ESM.docx]

**Supplementary Information (Text and Figures)**

Scripts used for keyword counting of SOTU and PBM transcripts are provided in Silver (2019). The keyword counts are available in Silver & Quigley (2019). Below is the list of keywords that match when the search is applied to a dictionary file containing over 99,000 US English words:

**energy:** 'energy'; **tax:** 'nontaxable', 'overtax', 'overtaxed', 'overtaxes', 'overtaxing', 'surtax', 'surtaxed', 'surtaxes', 'surtaxing', 'surtaxs', 'tax', 'taxable', 'taxation', 'taxations', 'taxed', 'taxes', 'taxing', 'taxpayer', 'taxpayers', 'taxs'; **defense:** 'defend', 'defense'; **education:** 'education'; **employment:** 'employ', 'employable', 'employe', 'employed', 'employee', 'employees', 'employer', 'employers', 'employes', 'employing', 'employment', 'employments', 'employs', 'underemployed', 'unemployable', 'unemployed', 'unemployeds', 'unemployment', 'unemployments'; **research:** 'research', 'researched', 'researcher', 'researchers', 'researches', 'researching', 'researchs'; **shooting:** 'shooting'; **space:** 'space'; **nuclear:** 'nuclear'; **natural resources:** 'natural resources'; **racism:** 'racism', 'civil rights'; **crime:** 'crime', 'crimes', 'criminal', 'criminally', 'criminals', 'decriminalization', 'decriminalizations', 'decriminalize', 'decriminalized', 'decriminalizes', 'decriminalizing'; **environment:** 'environment', 'environmental', 'environmentalism', 'environmentalisms', 'environmentalist', 'environmentalists', 'environmentally', 'environments'; **religion:** 'faith', 'god', 'prayer', 'religion'; **health:** 'health', 'healthful', 'healthfully', 'healthfulness', 'healthfulnesss', 'healthier', 'healthiest', 'healthily', 'healthiness', 'healthinesss', 'healths', 'healthy', 'unhealthful', 'unhealthier', 'unhealthiest', 'unhealthy'; **terror:** 'terror', 'terrorism', 'terrorisms', 'terrorist', 'terrorists', 'terrorize', 'terrorized', 'terrorizes', 'terrorizing', 'terrors'; **war:** 'war', 'warrior', 'warriors', 'wars'; **economy:** 'economic', 'economical', 'economically', 'economics', 'economicss', 'economy', 'economys', 'microeconomics', 'microeconomicss', 'socioeconomic', 'uneconomic', 'uneconomical'; **jobs:** 'jobs'; **business:** 'agribusiness', 'agribusinesses', 'agribusinesss', 'business', 'businesses', 'businesslike', 'businessman', 'businessmans', 'businessmen', 'businesss', 'businesswoman', 'businesswomans', 'businesswomen'; **drugs:** 'drugs', 'narcotics'; **inflation:** 'inflation'; **climate:** 'climate'; **science:** 'science', 'sciences', 'scientific', 'scientifically', 'scientist', 'scientists'; **gun:** 'gun', 'gunfire', 'gunman', 'guns', 'handgun', 'rifle', 'shotgun'; **tech:** 'biotechnology', 'biotechnologys', 'technical', 'technological', 'technologically', 'technologies', 'technologist', 'technologists', 'technology', 'technologys'; **military:** 'military'; **security:** 'security'; **housing:** 'housing'; **pollution:** 'pollution'

Two extra phrases, which do not appear in the dictionary file, are added to the list: 'civil rights' (under the 'racism' keyword) and 'natural resources' (under the 'natural resources' theme). The dictionary file used is a standard file among Linux systems, and the version used was provided with version 7.1-1 of the Ubuntu 'wamerican' package.

**
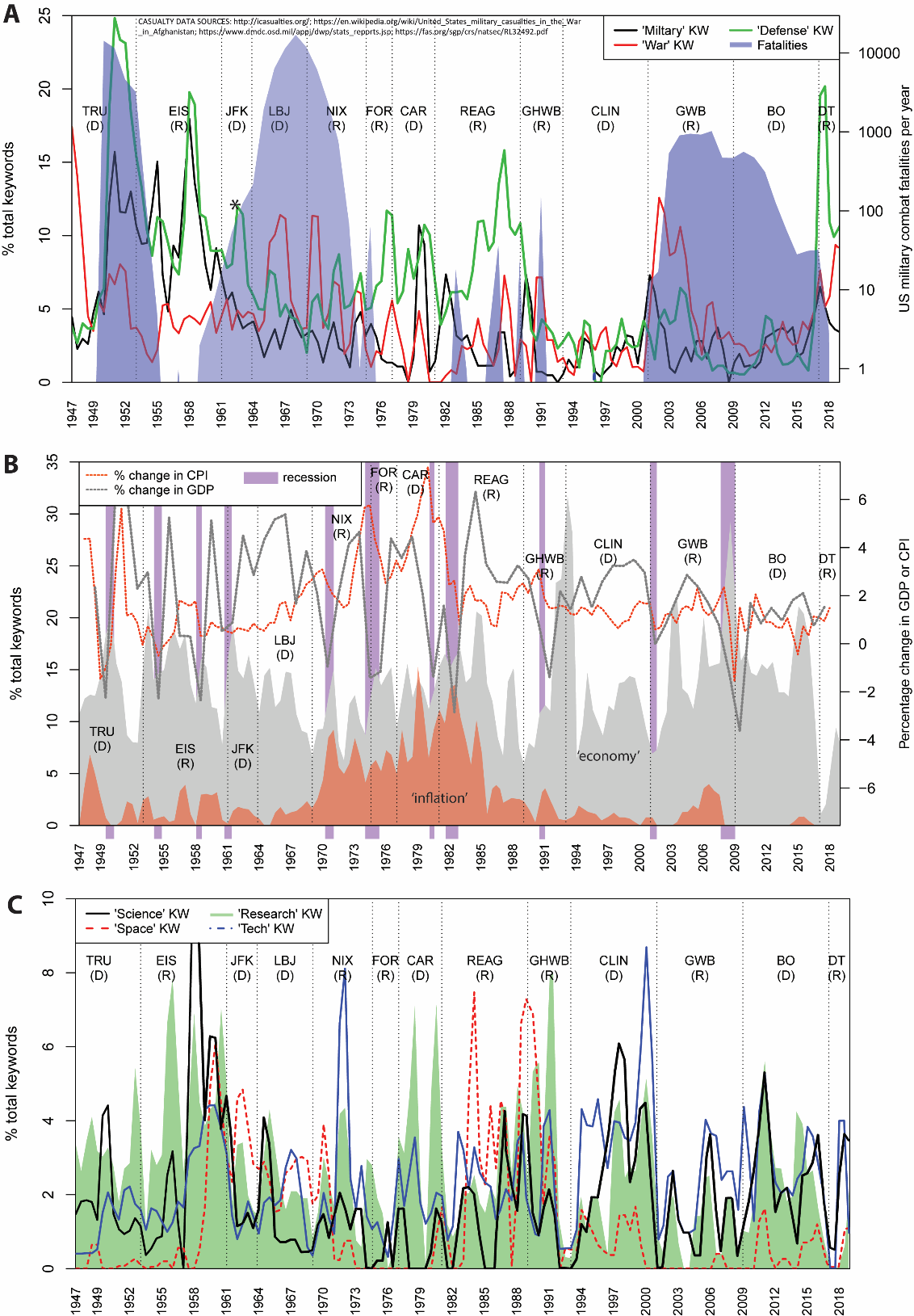
**

**Fig. S1. Examples of time-series of average keyword utility % versus significant military and socio-economic data. (A)** Time-series of ‘military’, ‘war’, and ‘defense’ average % keyword utility in SOTU and PBMs plotted with the log of U.S. military combat casualties per year (SOURCES: <http://icasualties.org/>; <https://en.wikipedia.org/wiki/United_States_military_casualties_in_the_War_in_Afghanistan>; <https://www.dmdc.osd.mil/appj/dwp/stats_reports.jsp>; <https://fas.org/sgp/crs/natsec/RL32492.pdf>). Keyword utility lines correspond to a 2-pt moving average of SOTU and PBMs, for distinct values see Fig. 1. Symbol * denotes the timing of Cuban Missile Crisis. Light grey lines denote presidential inauguration dates. **(B)** Time-series of ‘inflation’ and ‘economy’ keyword % utility (lines), shown with annual % change in U.S. Consumer Price index (CPI) and U.S. real gross domestic product (GDP) per capita (shaded areas), and timing of economic recessions (purple bars). Sources of CPI and GDP data shown in top right of figure. **(C)** Time-series of ‘science’, ‘space’, ‘technology’ (lines) and ‘research’ (shaded) % keyword utility averaged over the SOTU and PBMs.


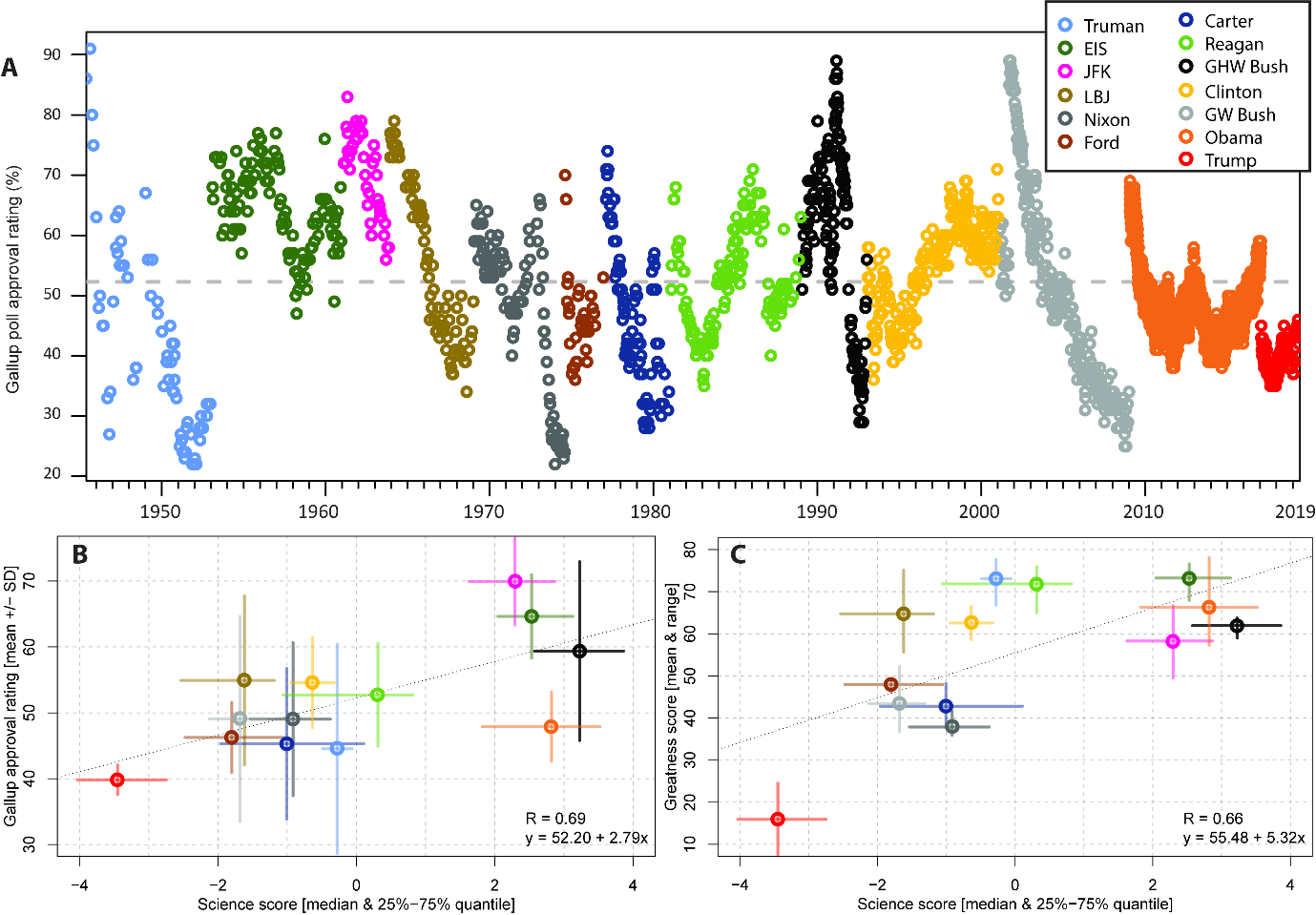


**Fig. S2** Time-series of presidential approval % Gallup poll data from 29 May 1945 to 16 June 2019. Gallup poll data was obtained from https://news.gallup.com/interactives/185273/presidential-job-approval-center.aspx (last accessed 20 June 2019). The y-axis is the Gallup poll % of survey participants that answered “Approve” to the question "*Do you approve or disapprove of the way [president’s name] is handling his job as president?*” in the corresponding poll survey. The horizontal grey dashed line shows the average approval rating for the sampled Presidents.
